# Diagnosis and treatment of a rare small intestine duplication in adult under double-balloon enteroscopy and laparoscopy

**DOI:** 10.1055/a-2299-2595

**Published:** 2024-04-29

**Authors:** Xing Xiong, Yong Tian, Dandan Zhao, Shusen Qian, Hongmei Ran, Tao Pan, Yihan Ma

**Affiliations:** 1 159418 Department of Gastroenterology and Hepatology, Chengdu First Peopleʼs Hospital, Chengdu, China; 2 118385 Clinical Medical College, Chengdu University of Traditional Chinese Medicine, Chengdu, China

A 35-year-old man was admitted to the hospital due to unexplained recurrent abdominal pain and hematochezia for 1 year. Lab results showed mild anemia (HGB 122 g/L). Abdominal enhanced computed tomography showed a blind tube-like structure near the right lower abdomen and ileum. The distal local wall was nodular, thickened, and significantly enhanced ( Fig. 1 ). Double-balloon enteroscopy (DBE) was then performed through the oral route and the anal route ( Video 1 ). A double lumen opening of the ileum was displayed approximately 1.2 m from the anal route. One irregular semi-circular ulcer with a white coating was found near the stricture in one lumen. It was suspected to be a small intestine duplication anomaly.

**Fig. 1:**
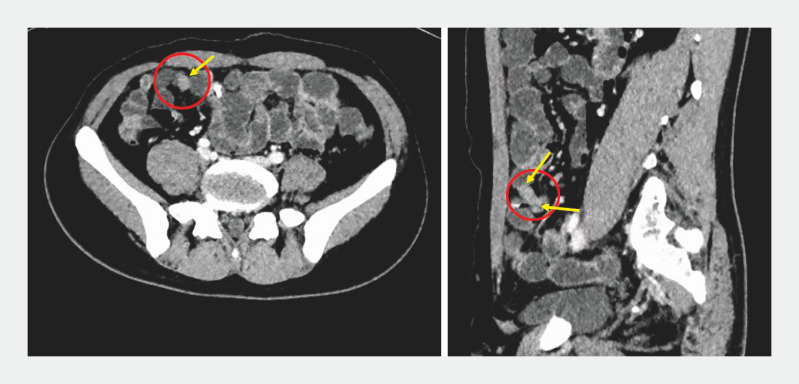
Computed tomography images showed a blind tube-like structure near the right lower abdomen and ileum.

Double-balloon enteroscopy and laparoscopic observation of ileal tubular duplication deformity with independent mesentery and blood supply.Video 1

During laparoscopic exploration ( Video 1 ), a lumen approximately 8 × 2 cm in size could be seen at the distal end of the ileum, approximately 30 cm away from the ileocecal region. Its mesentery showed a tubular lumen, which was different from Meckelʼs diverticulum. In particular, this tubular lumen had an independent mesentery and blood supply. Subsequently, we pulled out the ileum and used a cutting stapler to remove the duplicate deformed intestinal segment. The postoperative diagnosis was ileal duplication deformity ( Fig. 2 ). Pathology showed that intestinal mucosa contained ectopic gastric glands ( Fig. 3 ). The patient was discharged 9 days after surgery and did not experience any particular discomfort.

**Fig. 2:**
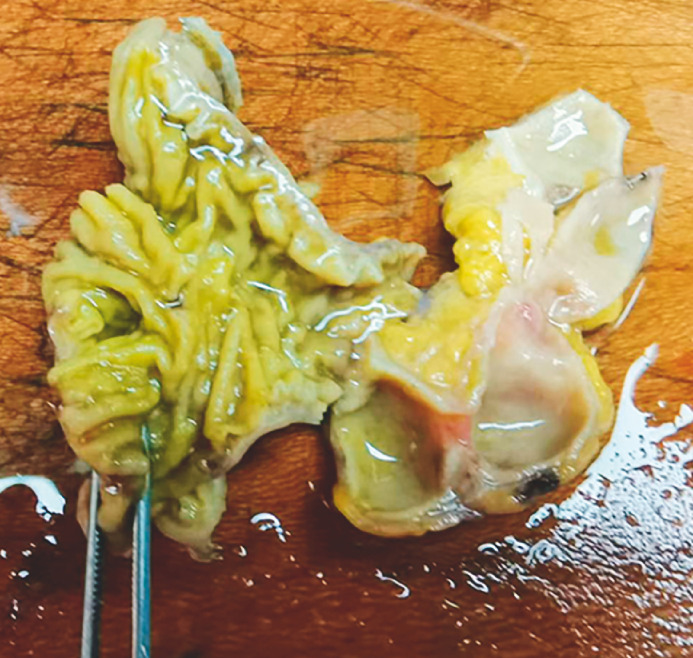
Duplicated and deformed small intestine after dissection.

**Fig. 3:**
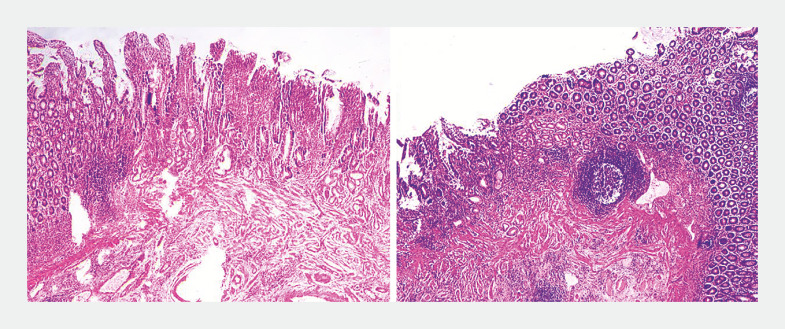
Pathological result indicated that intestinal mucosa contained ectopic gastric glands.

Intestinal duplication is a rare congenital anomaly that typically occurs during fetal or pediatric development 1 2 . Duplicated segments usually share a common wall and blood supply with native intestine. Clinical symptoms can manifest as abdominal pain, bloody stools, and even intestinal obstruction. It can easily be misdiagnosed as Meckelʼs diverticulum. For treatment, surgical intervention is required to correct deformities and restore normal function. Previous reports of small intestine duplication mainly occurred in children 1 3 . Here, we report a rare case of ileal tubular duplication deformity with an independent mesentery and blood supply in an adult male.

Endoscopy_UCTN_Code_CPL_1AM_2AF

## Notes

Endoscopy E-Videos https://eref.thieme.de/e-videos
*E-Videos* is an open access online section of the journal *Endoscopy* , reporting on interesting cases and new techniques in gastroenterological endoscopy. All papers include a high-quality video and are published with a Creative Commons CC-BY license. Endoscopy E-Videos qualify for HINARI discounts and waivers and eligibility is automatically checked during the submission process. We grant 100% waivers to articles whose corresponding authors are based in Group A countries and 50% waivers to those who are based in Group B countries as classified by Research4Life (see: https://www.research4life.org/access/eligibility/ ). This section has its own submission website at https://mc.manuscriptcentral.com/e-videos .
